# Isolated Ovarian Metastasis from Pancreatic Cancer Mimicking Primary Ovarian Neoplasia: Role of Molecular Analysis in Determining Diagnosis

**DOI:** 10.1089/pancan.2021.0001

**Published:** 2021-10-14

**Authors:** Catherine M. Tucker, Cheryl L. Godcharles, Wei Jiang, Charles J. Yeo, Norman G. Rosenblum, Ethan J. Halpern, William E. Luginbuhl, Anthony J. Prestipino

**Affiliations:** ^1^ Department of Pathology, Anatomy, and Cell Biology, Thomas Jefferson University Hospital, Philadelphia, Pennsylvania, USA.; ^2^ Department of Obstetrics and Gynecology, Thomas Jefferson University Hospital, Philadelphia, Pennsylvania, USA.; ^3^ Department of Pathology, Anatomy, and Cell Biology, Thomas Jefferson University, Philadelphia, Pennsylvania, USA.; ^4^ Section of Hepatopancreatobiliary Surgery, Department of Surgery, Thomas Jefferson University Hospital, Philadelphia, Pennsylvania, USA.; ^5^ Division of Gynecologic Oncology, Department of Obstetrics and Gynecology, Thomas Jefferson University Hospital, Philadelphia, Pennsylvania, USA.; ^6^ Department of Radiology, Thomas Jefferson University Hospital, Philadelphia, Pennsylvania, USA.; ^7^ Division of Hematology and Oncology, Department of Medicine, University of Pennsylvania, West Chester, Pennsylvania, USA.; ^8^ Department of Pathology, Anatomy, and Cell Biology, Thomas Jefferson University Hospital, Philadelphia, Pennsylvania, USA.

**Keywords:** pancreatic adenocarcinoma, KRAS G12R mutation, ovarian metastasis, mucinous tumor, molecular diagnostics, pathology

## Abstract

**Background and Presentation:** In this study, we present the case of a 64-year-old female with a chief complaint of abdominal pain and bloating, which had been persistent over a period of 4 months. Imaging revealed a 6.1-cm left-sided pancreatic mass as well as a 19.1-cm multiloculated cystic lesion in the pelvis, later revealed to be replacing the left ovary. The pancreatic mass was biopsied through endoscopic ultrasound-guided fine needle aspiration, and diagnosed as adenocarcinoma by cytology. The patient was treated with neoadjuvant chemotherapy and radiation before laparotomy for resection of the pancreas and left adnexal mass. Her response to treatment was followed radiologically and biochemically with cancer antigen (CA) 19-9 (114–35 U/mL), carcinoembryonic antigen (12–4.8 ng/mL), and CA-125 (119–15.3 U/mL) levels. She subsequently underwent an Appleby procedure, and resection of left pelvic mass and bilateral oophorectomy. Permanent sections revealed residual pancreatic ductal carcinoma with treatment effect, and a multicystic epithelial neoplasia of the left ovary for which the differential was primary ovarian carcinoma versus metastatic disease.

**Conclusions:** Molecular mutational analysis was performed on sections of both the ovarian tumor and the pancreatic tumor to aid in diagnosis. The ovarian tumor in this case showed exactly the same mutations, *KRAS G12R* and *TP53 G245S*, as in the treated pancreatic cancer. This raised the high probability that these tumors originated from the same clonal event. The findings suggested that the ovarian tumor was an isolated metastasis of the pancreatic primary, despite the morphologic ambiguity between the two sites of neoplasia.

## Clinical Course and Pathology

A 64-year-old female with a past medical history of tobacco abuse (∼24 pack-years) and fibroids and a past surgical history of total abdominal hysterectomy and bilateral salpingectomy presented to her primary care physician complaining of a 4-month history of progressive abdominal pain and distension. Magnetic resonance imaging revealed a 19.1-cm multiloculated cystic lesion filling the pelvis, most likely arising from one of the adnexal regions, and a 6.1-cm mass within the pancreatic tail, consistent with a locally advanced left-sided pancreatic cancer. The pancreatic mass was biopsied through endoscopic ultrasound-guided fine needle aspiration (FNA), and the patient was diagnosed with adenocarcinoma. She was then started on neoadjuvant chemotherapy for pancreatic adenocarcinoma.

**FIG. 1.**
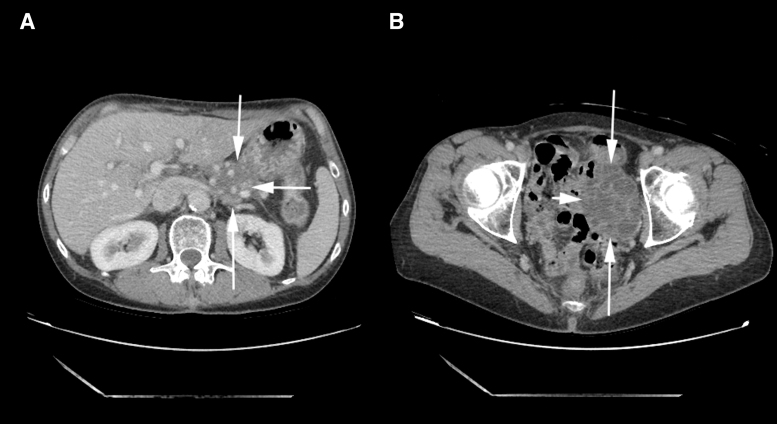
Computed tomography abdomen and pelvis showing the pancreatic and ovarian masses. **(A)** Mass lesion (5.9 × 3.6 cm) replacing the distal body and tail of the pancreas (*arrows*) with encasement of the adjacent vascular structures. The mass is inseparable from the posterior wall of the stomach and the left adrenal gland. **(B)** Cystic mass (6.9 × 5.6 cm) in the left adnexal region (*arrows*).

Follow-up imaging revealed a minimal decrease in size of the invasive mass within the pancreatic body and tail to 5.9 cm (Fig. 1A). The pelvic mass, however, appeared to have enlarged, with extension into the abdomen, in keeping with malignancy (Fig. 1B).

Treatment response was additionally monitored with cancer antigen (CA) 19-9 levels, which ranged from a high of 114 U/mL on initial presentation to 35 U/mL after treatment with neoadjuvant chemotherapy. The patient's carcinoembryonic antigen (CEA) decreased from 12 ng/mL on initial presentation to 4.8 ng/mL, and her CA-125 decreased from 119 to 15.3 U/mL. The CA-125/CEA ratio was 9.9 on initial presentation, which was less than 25 and therefore consistent with possible gastrointestinal/hepatobiliary origin, not ovarian origin.^1^

The patient was referred to our institution after initiation of neoadjuvant treatment, for discussion of surgical options in her treatment plan for her locally advanced adenocarcinoma of the body and tail of the pancreas and pelvic mass. Computed tomography of the abdomen/pelvis with contrast at her consultation visit revealed a pancreatic tumor with apparent encasement of adjacent vascular structures, including the celiac and superior mesenteric arteries and their branches. The differential for the pelvic mass was metastatic disease versus a primary ovarian process.

The patient then underwent additional chemotherapy, ultimately completing 10 cycles of combination chemotherapy with fluorouracil, leucovorin, irinotecan, and oxaliplatin (FOLFIRINOX), followed by external beam radiation therapy with concurrent oral Xeloda. Her postradiation therapy CA 19-9 was 9 U/mL, and CEA was 1.6 ng/mL. Follow-up imaging showed that the mass lesion involving the body to tail of the pancreas was minimally decreased in size from prior. The pelvic mass had decreased in size, but with an increased number of small cystic foci. Based on clinical, biochemical, and radiologic findings, an Appleby procedure was planned for resection of the pancreatic mass, as well as resection of the pelvic mass and bilateral oophorectomy. The decision was made to move forward with the operation after discussion with the patient, given the opportunity for safe resection of the pancreatic and pelvic masses.

The patient underwent distal pancreatectomy and *en bloc* splenectomy with *en bloc* resection of a portion of the common hepatic artery, and resection of the left pelvic mass and bilateral oophorectomy. Intraoperatively, exploration of the duodenum and pancreas revealed a firm mass involving the pancreatic body and tail, with invasion into the retroperitoneum. The common hepatic artery at the superior margin of the pancreatic body appeared to be involved. The superior mesenteric artery vessels were easily dissected free intraoperatively and did not appear to be grossly encased by tumor. On exploration of the pelvis, a large 10+ cm left cystic adnexal mass was identified, which was adherent to the left pelvic sidewall and also to a portion of sigmoid colon. Both left and right ovaries were removed.

**FIG. 2.**
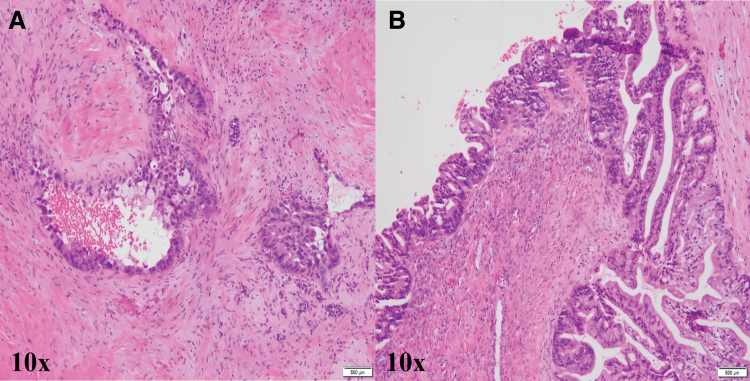
Histologic sections of the pancreatic and ovarian masses. **(A)** Permanent sections of the pancreatic mass showed a few residual viable groups of poorly differentiated adenocarcinoma, with evidence of extensive treatment effect (10 × objective). **(B)** Sections of the enlarged left ovary showed multiple neoplastic cysts, which were lined by intestinal type epithelial cells that showed stratification (10 × objective).

Gross examination of the distal pancreas revealed a 4.5-cm ill-defined mass in the pancreatic tail, located ∼2.5 cm from the pancreatic neck margin. Permanent sections (Fig. 2A) showed only a few residual viable groups of poorly differentiated adenocarcinoma, morphologically consistent with pancreatic ductal carcinoma (1.0 cm in greatest dimension). There was evidence of extensive treatment effect with multiple pools of acellular mucin in the area of the pancreatic tumor.

The residual viable pancreatic neoplastic cells stained strongly for cytokeratin (CA) 7, CA 19-9, and showed very weak scattered nuclear positivity for CDX2. The residual viable pancreatic neoplastic cells were negative for CK20, PAX8, and SATB2. Although not a specific immunostaining pattern, this was considered consistent with a primary pancreatic neoplasm.^2^ Margins of surgical resection, including proximal pancreatic parenchymal margin and peripancreatic tissue, were microscopically free of viable malignant neoplasia. Ten regional lymph nodes were negative for metastatic disease and showed no evidence of intranodal therapeutic effect. The final pathologic stage was pT1cpN0 (American Joint Committee on Cancer, 8th edition).

Gross examination of the left ovary consisted of a 206 g, 11 × 8.5 × 4.5 cm multiloculated ovarian mass with both solid and cystic areas. Multicystic areas were composed of smooth-walled cysts containing both serous and mucoid material. Microscopic sections of the enlarged left ovary (Fig. 2B) showed multiple neoplastic cysts lined by intestinal type epithelial cells that showed stratification, including foci of cribriform architecture. The tumor showed a multinodular growth with some of the neoplastic cells being far away from other groups of neoplastic glands, which is not typically seen in primary ovarian carcinomas.^3–7^ There was a range of cytologic and architectural atypia associated with the tumor as one frequently sees with metastatic pancreaticobiliary carcinomas. There was additionally an associated desmoplastic reaction around some neoplastic glands, which is rarely seen in primary carcinomas of the ovary, but more common in metastases.^4^ There was also a focus suggestive of surface involvement in one section, another feature that would support the diagnosis of metastatic carcinoma.^3,5–7^ The right ovary showed no evidence of neoplasia.

The neoplastic epithelial cells marked strongly and diffusely positive for CK7, showed positivity for CA 19-9, MUC1, and were diffusely positive for the intestinal markers CK20 and CDX2. The neoplastic cells were negative for MUC2, SATB2, CA-125, estrogen receptor, and PAX8. Mucinous ovarian neoplasms of the intestinal type have previously been found to express intestinal type markers.^8^ The pathologic differential diagnosis included borderline mucinous tumor of the ovary, intestinal type, showing multiple scattered foci of intraepithelial carcinoma and focal microinvasion versus isolated metastasis from the patient's known pancreatic primary.

A pan-cancer molecular mutational panel was then performed separately on sections from the ovarian and the pancreatic tumor to aid in diagnosis. This panel is performed in-house at our institution, and provides comprehensive detection of somatic mutations in 30 important cancer-related genes (*AKT1*, *ALK*, *BRAF*, *CTNNB1*, *DDR2*, *EGFR*, *EIF1AX*, *ERBB2*, *FGFR1*, *FGFR2*, *FGFR3*, *GNAS*, *HRAS*, *IDH1*, *IDH2*, *KIT*, *KRAS*, *MAP2K1*, *MET*, *NRAS*, *PDGFRA*, *PIK3CA*, *PTEN*, *RET*, *ROS*, *SMAD4*, *STK11*, *TERT*, *TP53*, and *TSHR*). The test is performed on DNA extracted from formalin-fixed, paraffin-embedded tumor tissue or FNA biopsy specimens. The Archer VariantPlex Comprehensive Thyroid and Lung Panel is used to perform DNA sequencing, in conjunction with the *SMAD4* gene, which uses anchored multiplex polymerase chain reaction to amplify regions of interest in the 30 genes listed. Amplicons are sequenced on an Illumina NextSeq next-generation sequencer. The ovarian tumor in this case showed exactly the same mutations, namely, *KRAS G12R* and *TP53 G245S*, as in the treated pancreatic cancer. This raised the high probability that these tumors originated from the same clonal event. The findings indicated that the ovarian tumor was an isolated metastasis of the pancreatic primary, despite the morphologic ambiguity between the two sites of neoplasia. Additional molecular testing by Perthera confirmed that the genomic profile in the left ovary was consistent with metastasis from a pancreatic primary. Outside testing further identified an inactivating mutation of *CDKN2A* and amplification of *GATA6*.

The patient is currently 15 months postoperation. Although she has improved significantly, her follow-up imaging and laboratory results are concerning for recurrent disease. Restaging imaging studies show an area of right upper quadrant omental infiltration (2.6 × 1.4 cm) and additional subtle abnormalities. Her CA 19-9 level has risen to 77 U/mL. There is no indication for surgery at this point. There was discussion of additional treatment with chemotherapy, but given the patient's difficult clinical course and a lack of data, the patient will instead follow up closely with medical oncology.

## Patient Consent Statement

Informed patient consent was obtained by the authors before publication of this article.

## Discussion

Pancreaticobiliary tumors with mucinous morphology that metastasize to the ovary are uncommon, accounting for nearly 14% of all metastatic mucinous tumors to the ovary.^3,9^ The case presented in this study was diagnostically challenging in that the metastatic pancreatic carcinoma mimicked a primary ovarian neoplasia morphologically, and the entities were not definitively distinguishable by immunohistochemistry alone. Histologic sections of the ovarian mass revealed neoplasia highly reminiscent of primary intestinal type mucinous borderline carcinoma of the ovary.

Also supporting the possibility of a primary ovarian neoplasm was the fact that the mass was unilateral, as the right ovary was shown to be unaffected on histologic examination. It is well established that a significant proportion of cases of metastatic pancreatic adenocarcinoma to the ovary present bilaterally.^3,9,10^ In fact, the case presented in this study represents an exception to algorithms derived by previous and more recent studies using tumor size and laterality to accurately classify a substantial portion of ovarian mucinous tumors as either primary or secondary. Previous studies have shown that bilateral tumors of any size, or a unilateral tumor <10 cm likely represents metastatic disease, while a unilateral tumor ≥10 cm likely represents primary disease. This original algorithm correctly classified 84% of tumors overall in Yemelyanova et al.'s study of 194 tumors, including 95% of pancreaticobiliary primaries. They optimized the algorithm by adjusting the size criterion to 13 cm, which correctly classified 100% of their pancreaticobiliary primaries, but still would not correctly classify our case, as it was 14 cm at its smallest around the time of the patient's initial presentation.^9^

There were, however, subtle clues supportive of metastasis from the patient's primary pancreatic tumor. Primary ovarian carcinomas typically do not show the multinodular growth pattern seen in our case, or desmoplasia around neoplastic glands, both of which are more often seen in metastases as opposed to primary ovarian carcinomas.^3–7^ There was also a range of cytologic and architectural atypia associated with the tumor, which is often seen with metastatic pancreaticobiliary carcinomas. Finally, there was also a focus suggestive of surface involvement in one section, another feature that would support the diagnosis of metastatic carcinoma.^3,5–7^

Molecular mutational analysis was key to ultimately diagnosing the left ovarian mass as metastatic pancreatic adenocarcinoma. The ovarian tumor in this case showed exactly the same mutations, *KRAS G12R* and *TP53 G245S*, as in the treated pancreatic cancer, highly suggestive of the tumors originating from the same clonal event. Of note, the *KRAS G12R* mutation is rare in many cancer types (<3% overall) such as lung and colorectal cancers (∼1%), yet is the third most common *KRAS* mutation in pancreatic ductal adenocarcinoma (comprising 16% of all *KRAS* mutations).^11,12^ In addition, although *KRAS G12R* mutation has been identified rarely in ovarian cancers (0.7% of 142 total cases of epithelial ovarian carcinoma investigated by Rechsteiner et al.), the combination of the exact same *TP53* mutation in both tumors would strongly suggest this being a metastasis rather than a synchronous primary.^13^ The use of molecular analysis to distinguish primary ovarian carcinoma from pancreatic metastasis has not been described extensively in the literature. Lowery et al. did use the identification of two distinct *KRAS* mutations in a primary pancreatic ductal adenocarcinoma (G12V) and a synchronous ovarian mucinous neoplasm (G12D) to successfully determine, in contrast to our case, that the ovarian tumor was indeed an ovarian primary.^14^ Of note, the rate of *KRAS* mutation does not differ significantly between tumors with widespread metastases, in contrast to the *DPC4* gene, which was inactivated more frequently in metastatic disease.^15^ Loss of *DPC4* has previously been considered a useful immunohistochemical marker for establishing pancreaticobiliary tract origin in metastatic mucinous carcinoma of the ovary, as primary ovarian mucinous tumors have not been shown to exhibit loss of expression.^3^

Further supporting the final diagnosis was the radiologic decrease in size of the ovarian neoplasm secondary to neoadjuvant treatment, and the fact that the patient's CA-125/CEA ratio was 9.9. Levels of the CA-125/CEA ratio less than 25 are associated with metastatic neoplasia to the ovary.^1^

## Conclusions

The case presented in this study represents a diagnostic challenge clinically, radiologically, and pathologically. The patient presented with the pancreatic and left pelvic mass simultaneously, and the left pelvic mass was considered to be “almost certainly” of ovarian origin radiologically. As described above, histologic sections were highly reminiscent of borderline mucinous tumor of the ovary, intestinal type, morphologically, and not definitively distinguishable from pancreatic adenocarcinoma with immunohistochemical staining. Although subtle morphologic findings and biochemical markers supported the fact that this was a pancreatic metastasis, molecular analysis was indispensible in rendering the final diagnosis in this patient.

## Abbreviations Used

CA: cancer antigen
CEA: carcinoembryonic antigen
CK: cytokeratin
FNA: fine needle aspiration
